# New Checklist for the Heuristic Evaluation of mHealth Apps (HE4EH): Development and Usability Study

**DOI:** 10.2196/20353

**Published:** 2020-10-28

**Authors:** Kamran Khowaja, Dena Al-Thani

**Affiliations:** 1 Information & Computing Technology College of Science & Engineering Hamad Bin Khalifa University Doha Qatar

**Keywords:** mHealth, eHealth, heuristic evaluation, expert evaluation, self-monitoring, behavior change, design guidelines, framework

## Abstract

**Background:**

Diabetes is one of the leading causes of death in developing countries. Existing mobile health (mHealth) app design guidelines lack a description of the support of continuous self-monitoring of health status, behavior change to improve and adopt a healthy lifestyle, and communication with health educators and health care professionals in case of any need.

**Objective:**

This paper presents the development of a specialized set of heuristics called heuristic evaluation for mHealth apps (HE4EH) as an all-in-one tool and its applicability by performing a heuristic evaluation of an mHealth app.

**Methods:**

An extensive review of heuristics and checklists was used to develop the HE4EH. The HE4EH was evaluated by domain experts for heuristics, checklist items, severity ratings, and overall satisfaction. The OneTouch app, which helps individuals with diabetes manage their blood glucose levels, was evaluated using HE4EH to identify usability problems that need to be fixed in the app.

**Results:**

The expert evaluation of HE4EH revealed that the heuristics were important, relevant, and clear. The checklist items across the heuristics were clear, relevant, and acceptably grouped. In terms of evaluating the OneTouch app using the HE4EH, the most frequently violated heuristics included Content, Visibility, Match, and Self-monitoring. Most of the usability problems found were minor. The system usability scale score indicated that the OneTouch app is marginally acceptable.

**Conclusions:**

This heuristic evaluation using the OneTouch app shows that the HE4EH can play a vital role for designers, researchers, and practitioners to use HE4EH heuristics and checklist items as a tool to design a new or evaluate and improve an existing mHealth app.

## Introduction

The leading reason for morbidity and mortality, especially in developing countries, is chronic disease [1]. About 80% of these deaths in developing countries are due to cardiovascular disease and diabetes mellitus, a proportion that is higher than in developed countries. It is expected that this number will increase further to 85% by 2030. The World Health Organization has reported that the number of people with these diseases increased from 108 million in 1980 to 422 million in 2014 [2]. At least 17% of the population in all the Gulf countries, including Qatar (location of the workplace of both authors of this manuscript), has diabetes. Researchers at Weill Cornell Medicine-Qatar have predicted that the prevalence rate of type II diabetes will increase from 12% in 2012 to at least 24% by 2050 [3]. One main reported finding is that most cases are due to obesity. This shows a need to support affected individuals with health care education and lifestyle changes such as a healthy diet and increasing physically active to improve their quality of life. Beratarrechea and colleagues [1] conducted a systematic review on mobile health (mHealth) apps in developing countries and highlighted that mHealth apps address health care system constraints related to increased demand and limited resources. Developing countries have barriers, including limited health care human resources, insufficient finances, increasing populations, and an inability to reach the portion of the population living in remote areas, among others. Proper adherence to chronic disease management is essential to improve an individual’s quality of life and health-related outcomes [4]. Hamine and colleagues [4] also conducted a systematic review of the impact of mHealth on interventions and found that mHealth apps are increasingly accessible and highly acceptable tools for patient communication, monitoring, and education. They are also effective means to facilitate self-management. Other barriers, including language, literacy, access to a smartphone, cost, access to the internet, or mobile network, are typically present everywhere. This shows that there is a need to design mHealth tools with these barriers in mind so that targeted patients can easily use the developed mHealth solution with minimal training or support. To keep patients motivated in using mHealth solutions on a regular basis throughout the treatment or intervention, gamification is increasingly adopted, as it facilitates self-management of chronic conditions [5]. Thus, an app needs to support an individual to continuously self-monitor his or her health status, change behavior to improve his or her lifestyle, and communicate with health educators and health care professionals in case of any need.

Designers typically use an existing set of guidelines or heuristics as a base to design a new app; they also evaluate and improve the usability aspects of a developed app in light of an existing set of guidelines or heuristics. A search on guidelines and heuristics did not reveal an all-in-one package that supports self-monitoring, behavior change, and communication with health care professionals, as already mentioned, but it did reveal related guidelines and heuristics. These include mHealth apps [6], privacy of mHealth apps for self-tracking [7], electronic medical records [8,9], personal health records [10], eHealth [11], patient safety [12], and electronic health records [12]. To the best of our knowledge, there is no existing set of guidelines or heuristics that can be used to serve the purpose of evaluating an app that supports self-monitoring, behavior change, and communication with health care professionals.

Once an app is developed, a potential end user of the app or a domain expert can be recruited to evaluate the app. End users are typically exposed to the app towards the last stage of app development; therefore, the expertise of domain experts is utilized to quickly evaluate the app on behalf of end users. In this research, domain experts evaluated the OneTouch app. Methods that can be used for an expert evaluation include heuristic evaluation [13]; cognitive walkthrough [14,15]; goals, operators, methods, and selection [16]; keystroke-level model [17]; or using results of a previous study as the basis to prove or disprove different aspects of the design. Among these methods, heuristic evaluation is not only quick but also inexpensive and easy to perform in comparison to other methods.

This paper presents the development of a specialized set of heuristics based on existing heuristics, called the heuristic evaluation for mHealth apps (HE4EH), that can be used as a tool to design or evaluate an mHealth app. This paper is structured as follows. The Methods section presents the mHealth app design framework. The Results section presents the evaluation of the HE4EH by domain experts and its applicability to evaluate the OneTouch app. Last, the article is concluded in the Discussion section.

## Methods

### Compilation of Heuristics

**Figure 1:**
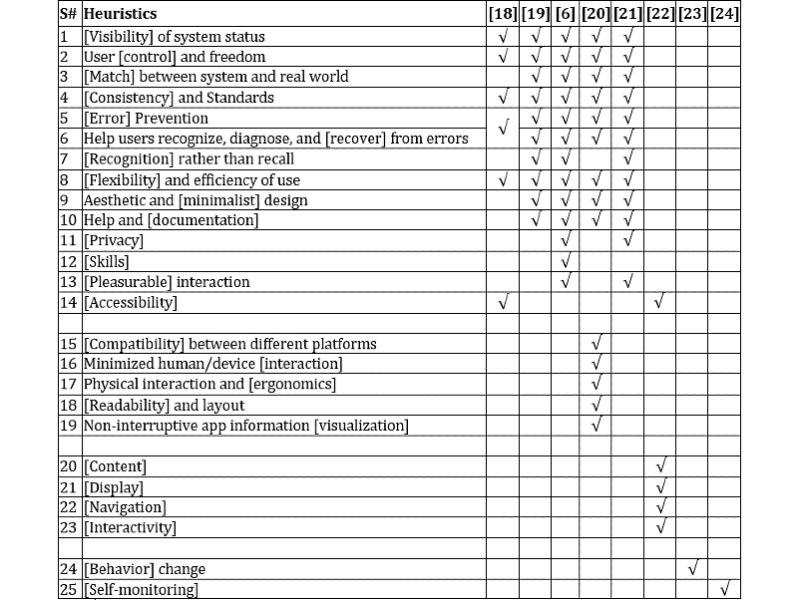
Compilation of heuristics.

To compile the heuristics, we searched for existing sets of mobile heuristics, mHealth heuristics, behavioral change, and self-monitoring of blood glucose in electronic databases, including Scopus, Web of Science, PubMed, and Google Scholar. The research revealed 8 sets, by Shneiderman [18], Nielsen [19], Gómez et al [6], Lacerda et al [20], Dourado and Canedo [21], Monkman and Kushniruk [22], Abraham and Michie [23], and the International Diabetes Federation [24]. Figure 1 presents the compilation of heuristics retrieved from the results. The single word from each heuristic that is written in square brackets is used as the shorter name of the heuristic in the following sections.

### Intraset Heuristics

There are similar heuristics across the sets; for instance, consistency and standards, strive for consistency, and consistency mean the same thing. Similarly, flexibility and efficiency of use, efficient interaction, customizability, and efficiency also mean the same thing. There are also other examples. Therefore, all such heuristics were grouped together using a concept similar to an affinity diagram.

### Interset Checklist Items

Once all the related heuristics were grouped, it became obvious that there would be multiple related checklist items. The next step was to identify all those related checklist items and group, merge, and expand the checklist items wherever needed. The checklist items were mostly in the form of statements. Each statement was converted into a question, so the checklist items could be used to identify which items satisfied, did not satisfy, and were not applicable for the app to support the researchers and practitioners in evaluating the mHealth app.

### mHealth Heuristics Framework

**Figure 2:**
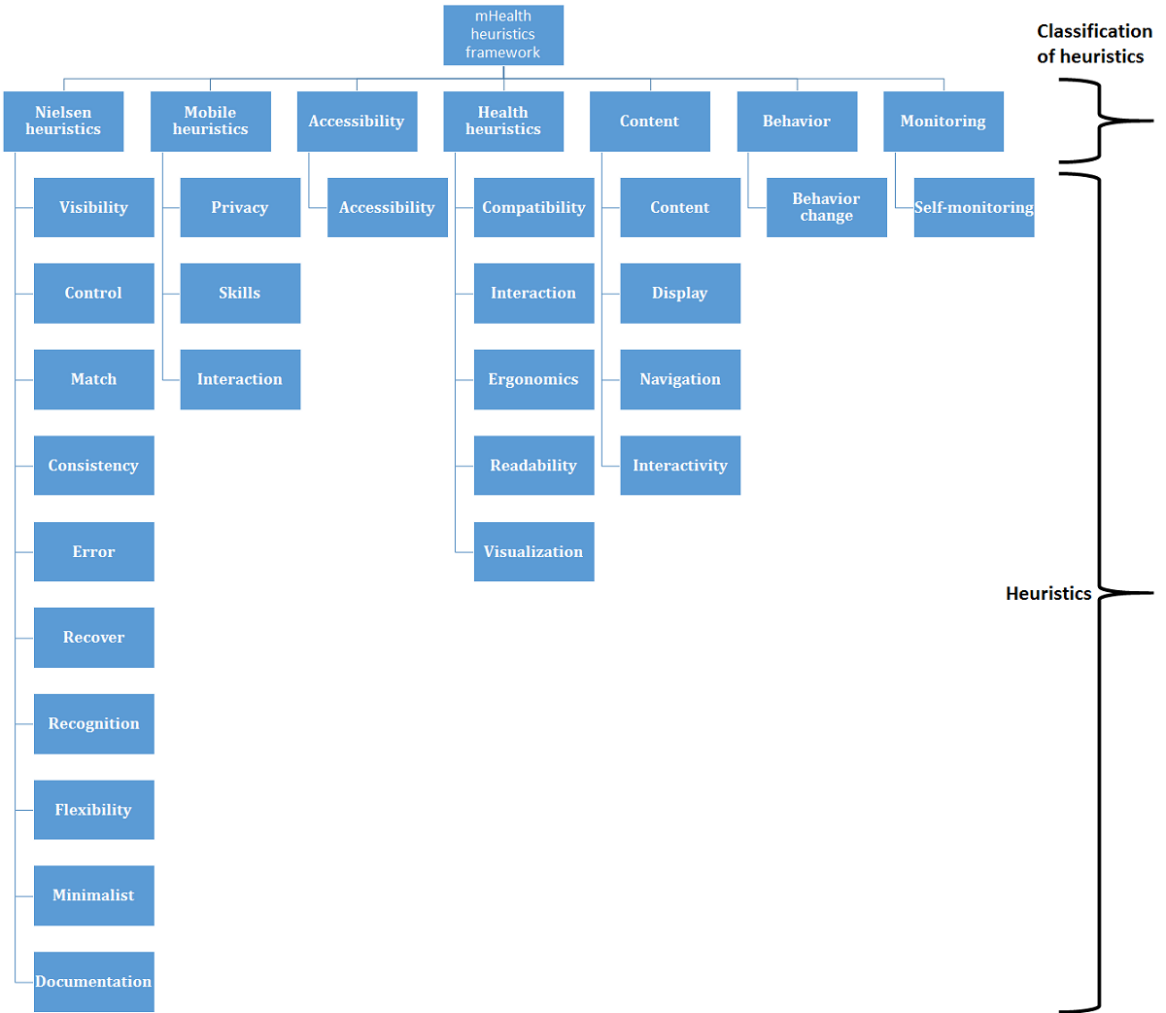
Mobile health (mHealth) heuristics framework.

Figure 2 shows the framework of an mHealth heuristics. The 25 heuristics in the set were classified into 7 types of heuristics. The framework consists of a set of components where each component contains specific details and the interconnection between the components form a structure of the framework. Similarly, the heuristics and their checklist items can be associated with the components and their details, respectively, and the interconnection between a group of heuristics to a type of heuristics forms a structure of the framework. Furthermore, the checklist items associated with each heuristic can be adapted to meet more or specific needs. Multimedia Appendix 1 presents the distribution of the heuristics and checklist items. An accessibility heuristic contains one checklist item only. However, there are various accessibility guidelines in the literature that can be used; the most used accessibility guidelines in recent years are the Web Content Accessibility Guidelines 1.0, 2.0, and 2.1. Similarly, in the context of this research, the checklist items of the self-monitoring heuristics are specific to diabetes; however, they can be adapted with a set of guidelines specific to any other chronic disease such as cancer, HIV, or cardiovascular disease or acute, but equally life-threatening, conditions such as ongoing coronavirus disease.

### Expert Evaluation Study 1

This subsection presents the overall study design of the expert evaluation study 1, while the results are presented in the Results section.

#### Participants and Recruitment

Experts are widely used in research studies to mainly gather their opinion and improve the instrument based on their feedback. Experts were also used in this research to improve the instrument (ie, a set of heuristics and checklist items). All the participants were recruited through convenience sampling and snowball sampling.

#### Instruments Used

The HE4EH is a proposed set of heuristics that contains 25 heuristics (see Figure 1) and a total of 436 checklist items (See Multimedia Appendix 1).

The prestudy questionnaire included questions related to the demographic information of the experts. The demographic questions included gender; occupation; industry; experience (in years); familiarity with types of diabetes; level of human-computer interaction (HCI), usability, user interface, or user experience design experience; experience working with patients with diabetes; and involvement in a number of projects for patients with diabetes.

The expert review questionnaire consisted of 4 parts, namely heuristics, checklist items, severity ratings, and satisfaction. A brief description of each follows.

The heuristics part of the expert review questionnaire presents all 25 heuristics provided to the experts; for each heuristic, the expert had to answer one question each for the importance of the heuristic, the relevancy of the heuristic, and clarity of the heuristic in the set. Each question was rated using a 5-point Likert scale. The items corresponding to an odd-numbered value for importance were “Not Important,” “Moderately Important,” and “Very Important”; similarly, the items corresponding to an odd-numbered value for relevancy included “Not Relevant,” “Moderately Relevant,” and “Very Relevant.” Last, the items corresponding with odd-numbered values for clarity were “Very Poor,” “Barely Acceptable,” and “Very Good.”

A total of 436 checklist items were presented to the experts in the expert review questionnaire. Similar to the heuristics, for each checklist item, the expert had to answer one question each for the clarity, grouping, and relevance of the checklist item. Each question for clarity, grouping, and relevance was rating using a 5-point Likert scale. The items associated with odd-numbered values for clarity included “Very Poor,” “Barely Acceptable,” and “Very Good”; similarly, the items associated with odd-numbered values for grouping were “Strongly Disagree,” “Undecided,” and “Strongly Agree.” Last, the items associated with odd-numbered values for relevance were “Not Relevant,” “Moderately Relevant,” and “Very Relevant.”

Researchers in this space typically use a 5-point severity rating scale where 0 means “No problem,” 1 means “Cosmetic,” 2 means “Minor,” 3 means “Major,” and 4 means “Catastrophe.” We decided to investigate 3 alternate severity rating scales. For the individual items in the 3 alternative scales, experts were asked to choose a suitable option on a 5-point Likert scale from “Very Difficult” to “Very Easy.” Then, we used descriptive statistics to identify the most suitable of the 3 scales.

The first alternative scale was scored as follows: 0, “No Violation”; 1, “Low”; 2, “Moderate”; 3, “High, Severe.” The second alternative scale was scored as follows: 0, “Not Applicable”; 1, “No Violation”; 2, “Minor”; 3, “Major” 4, “Catastrophe.” The third alternative scale was scored as follows: 0, “No Violation”; 1, “Low”; 2, “Medium”; 3, “High.”

The last part of the expert review evaluation asked the expert about their level of satisfaction in terms of using HE4EH for the evaluation of an mHealth app. They were asked to answer 13 questions (see Multimedia Appendix 2) using a 5-point Likert scale from 1 for “Strongly Disagree” to 5 for “Strongly Agree.”

#### Study Protocol

Each expert received a separate email to provide consent for the evaluation of a set of heuristics and the checklist items. In the email, they were briefly informed about the background of the study and its objectives and were introduced to the set of heuristics and its checklist items. The experts who agreed to participate in the study were sent another email with 3 instruments: (1) prestudy questionnaire, (2) heuristics and checklist items, and (3) expert review questionnaire. They were asked to complete the prestudy questionnaire first and then review all the heuristics and associated checklist items. Last, they were asked to complete a comprehensive expert review questionnaire and return the completed questionnaires via email.

#### Data Analysis

Microsoft Excel software was used to analyze the data collected in this study.

For each heuristic, checklist item, severity rating, and satisfaction rating, the expert was asked to answer one or more questions using a 5-point or 4-point Likert scale. The frequency was calculated as a sum of responses for each Likert-scale score for each question.

### Expert Evaluation Study 2

This subsection presents the overall study design of expert evaluation study 2, while the results are presented in the Results section.

#### Participants and Recruitment

**Figure 3:**
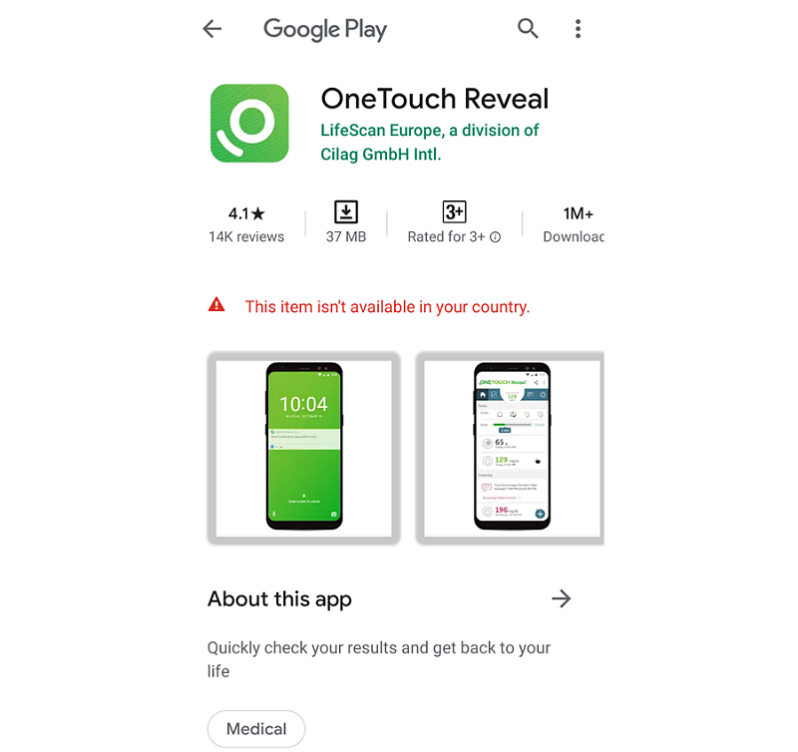
Error message for the app in the Google Play Store.

In studies evaluating heuristics, at least 3 participants are recommended, as they can contribute to identifying ≥75% of the usability problems in the app or the system used for the evaluation [13]. In this research, a total of 7 participants took part in the evaluation. The approach used to recruit participants was like that of expert evaluation study 1. Initially, 10 participants were recruited and divided into 2 sets of 5 groups, and only 1 participant was in the group. Three participants using an Android smartphone informed us they were unable to download the app. The Google Play Store was giving the following error message: “This item isn’t available in your country” as shown in Figure 3. This shows that access to the app was possibly restricted for certain countries; therefore, the participants from Malaysia and Pakistan could not download the app. It was then decided to proceed with the 7 participants from Qatar. The group-related details are described in Results: Expert Evaluation Study 2.

#### App Selection

Websites with rankings of apps to manage diabetes were searched. The selection criteria used to choose a webpage and the app were as follows: (1) webpage provided the names and descriptions of apps to manage diabetes, (2) app was available for both Android and iOS devices, (3) app was free to download, and (4) app did not have in-app purchases.

One of the pages entitled “The best diabetes apps of 2019” [25] was also part of the results. This webpage provided details for 13 apps, of which 3 apps met the aforementioned criteria, namely the Sugar Sense Diabetes app, OneTouch Reveal, and BeatO. The first app was excluded because it was not found in the Google Play Store. The iOS and Android ratings for the remaining 2 apps were 4.7 and 4.0, respectively, and 2.7 and 4.5, respectively. Based on the ratings for both platforms, the OneTouch app was selected for the evaluation in this research. The details of the OneTouch app are described in the next subsection.

#### Instruments Used

The proposed set of heuristic HE4ED and its checklist were used as the instruments in this research. The details of heuristics and checklists are discussed in detail in the earlier sections.

The OneTouch app helps manage blood glucose levels; this app complements the OneTouch Verio Flex meter. Once the blood glucose readings are added, the app automatically searches for and highlights trends and provides push notifications so the user can take the necessary actions. Physicians have access to their patients’ data; physicians can change their patients’ diet plans based on their history. The app also provides the opportunity to share data about progress with family members and friends. Furthermore, users can log in to their accounts online from their desktop or laptop. This app was carefully chosen because it may be useful for an individual with diabetes and obesity in the Middle East and North Africa region.

The prestudy questionnaire contained questions related to demographic information. The questions included gender, age, highest degree or level of education, designation (if working), HCI-related courses taken, previously evaluated apps, and set of heuristics or guidelines used.

The usability problem reporting form allowed participants to report identified usability problems with the OneTouch app. For each identified problem, the participant needed to report the broken heuristic and checklist, description of the problem, possible solution from their perspective, and severity rating. The severity rating ranged from 0 to 4 inclusive: “not a problem,” “cosmetic problem only,” “minor usability problem,” “major usability problem,” and “usability catastrophe.” The typical severity rating scale was chosen for 2 reasons. First, the experts’ opinions on the severity rating scales were almost the same. Second, the new ratings may not have given a better picture as each expert only received about 25% of the checklist items. Researchers conducting heuristic evaluation studies typically use the same severity scale mentioned in this subsection rather than the one proposed in this study.

The system usability scale (SUS) by Brooke [26] was used as the poststudy questionnaire. The usability measurements included in the SUS cover effectiveness, efficiency, and user satisfaction. The questionnaire includes 10 statements, and for each statement, the respondent needs to select the best possible choice based on a 5-point Likert scale from “strongly disagree” to “strongly agree.”

#### Study Protocol

We sent an email to the participants to brief them about the set of heuristics developed and the evaluation of the OneTouch app. They were informed that they would be divided into 5 groups, with 1 participant in each group, and groups would be randomly formed. They were asked to give consent by replying to the email. The participants consenting to participant were sent another email with the following:

- Set of instructions to download and install the OneTouch app on their Android or iOS device and to register an account for themselves. They were informed that they would evaluate the app on their own.
- Set of heuristics and either 107 or 108 checklist items (ie, about 25% of the total checklist items). We provided about one-fourth of the checklist items to limit workload and, at the same time, define a minimal set of checklist items that could be useful to evaluate the app. These checklist items were equally distributed in a way that each heuristic has one or more checklist item. There were some similarities in the checklists between groups.
- Pre-study questionnaire, usability problems reporting form for the OneTouch app, and poststudy questionnaire. They were informed to complete these in the following order: prestudy questionnaire, the evaluation of the OneTouch app and reporting of usability problems in the reporting form, and poststudy questionnaire. We requested that they return all completed files via email.

They were also informed that, in case of any confusion or query, they could always communicate via email, and a Skype call could be initiated for discussion, if needed. They were also asked to nominate a potential list of participants who would be interested in evaluating the app.

#### Data Analysis

Microsoft Excel software was also used to analyze the data collected during expert evaluation study 2.

For each heuristic, the usability problems represent the sum of usability problems found for all the severity ratings.

For each heuristic, the average severity rating represents the average of usability problems found for all the severity ratings.

The standard calculation method for the SUS score was used in this research.

## Results

### Expert Evaluation Study 1

Table 1 shows the demographic information of all the experts who participated in the study.

**Table 1:** Demographic information of the experts who participated in expert evaluation study 1.

Participant	Occupation	Industry	Experience (years)	HCI^a^ expertise	Projects^b^
Expert 1	Assistant professor	Education	18	Intermediate	N/A^c^
Expert 2	Senior software engineer	IT^d^	10	Intermediate	1
Expert 3	Assistant professor	Education	17	Intermediate	0
Expert 4	Associate professor	Education	20	Expert	2
Expert 5	Associate professor	Education	23	Expert	2

^a^HCI: human-computer interaction.

^b^Number of projects with patients with diabetes.

^c^N/A: not applicable.

^d^IT: information technology.

#### Heuristic

Multimedia Appendix 3 shows the expert opinions for the 3 questions (importance, relevance, and clarity of the heuristic) asked for all 25 heuristics of the set. The first column shows the serial number, while the second column shows the full name of the heuristic. The remaining 9 columns show the sum of the opinions for the aforementioned 3 questions. The sum of the expert opinions is shown in 3 columns for each question.

The numeric values in the 3 columns (from left to right) for the importance of a heuristic represent the number of responses for “Very Important,” “Important,” and “Moderately Important,” respectively. Similarly, the numeric values in the next 3 columns (from left to right) for the relevance of a heuristic represent the number of responses for “Very Relevant,” “Relevant,” and “Moderately Relevant.” The numeric values in the last 3 columns (from left to right) for the clarity of a heuristic represent the number of responses for “Very Good,” “Good,” and “Barely Acceptable.” There are only 3 negative responses for the “Little Importance” item of the Likert scale for a question related to the importance of the heuristic; the 3 heuristics marked with an asterisk “*” had 1 negative response each. Therefore, the 2 columns for the negative items on the Likert scale are not shown in the table. Similarly, 1 expert did not answer a question related to the importance and relevance of the heuristic for 3 heuristics; these heuristics are marked with a hash “#.”

Of the responses, 90.4% (113/125) showed that the heuristics are important, 4.8% (6/125) showed that they are moderately important, and 2.4% (3/125) showed that heuristics are of little importance, while the remaining 2.4% (3/125) were unanswered.

Of the responses, 93.6% (117/125) showed that the heuristics are relevant, and 4.0% (5/125) of the responses showed that they are moderately relevant, while the remaining 2.4% (3/125) of the responses were unanswered.

Of the responses, 74.4% (93/125) showed that the heuristics are clear, while the remaining 25.6% (93/125) showed that they are barely acceptable.

#### Checklist Items

Multimedia Appendix 4 shows the mean scores of all 3 questions (ie, clarity, grouping, and relevance) for each checklist item. The means are presented in ranges for each question. The analysis of the mean scores revealed that the minimum mean score for each question of the checklist was 2.8. Therefore, the ranges are presented in Multimedia Appendix 4 in increments of 0.2 (except the last range, which is an increment of 0.21) from 2.8 to 5.0. The decimal value of the checklist items represents the heuristic number in the set (whole number) and the checklist item for that heuristic (fractional number). The results of the questions are discussed in the following paragraphs.

The results show that the experts had a neutral opinion about the clarity of 2 of the 436 (0.5%) checklist items (ie, 3.26 and 17.4). The experts agreed for 173 of the 436 (39.6%) checklist items, while they strongly agreed for the remaining 262 of the 436 (60.0%) checklist items.

In terms of the grouping of checklists for each heuristic, the results show that the experts agreed for 149 of the 436 (34.1%) checklist items, while the experts strongly agreed for the remaining 288 of the 436 (66.0%) checklist items.

The results show that the experts had a neutral opinion about the relevance of 2 of the 436 (0.5%) checklist items (ie, 12.3 and 17.4). The experts agreed for 141 of the 436 (32.3%) checklist items, while they strongly agreed for the relevance of the remaining 294 of the 436 (67.4%) checklist items.

#### Severity Rating Scales

Multimedia Appendix 5, Multimedia Appendix 6, and Multimedia Appendix 7 present the results of 3 different severity rating scales in terms of the mean individual rating or score and the overall mean of all the ratings or scores. The results show that there is a subtle difference between all 3 rating scales.

#### Satisfaction

Multimedia Appendix 2 shows the level of satisfaction with using the HE4EH to evaluate an mHealth app. The numeric values in the 5 columns (from left to right) indicate the sum of the responses for “Strongly Agree,” “Agree,” “Neutral,” “Disagree,” and “Strongly Disagree” for each question.

According to the experts, sufficient or enough material was used to develop te HE4EH; it is not complex to use and provides useful information to design or evaluate an mHealth app. The terminologies used in the HE4EH are clear and easy to understand, learn, and use; the length of the HE4EH is suitable for the evaluation of mHealth apps that aim to improve the health of patients, especially those with diabetes in the context of this research, and allow them to self-monitor and change their behaviors. They were satisfied with the number of checklist items and their categorization, and they could use them to evaluate an app.

### Expert Evaluation Study 2

The demographic information of the recruited participants is presented in Table 2.

**Figure 4:**
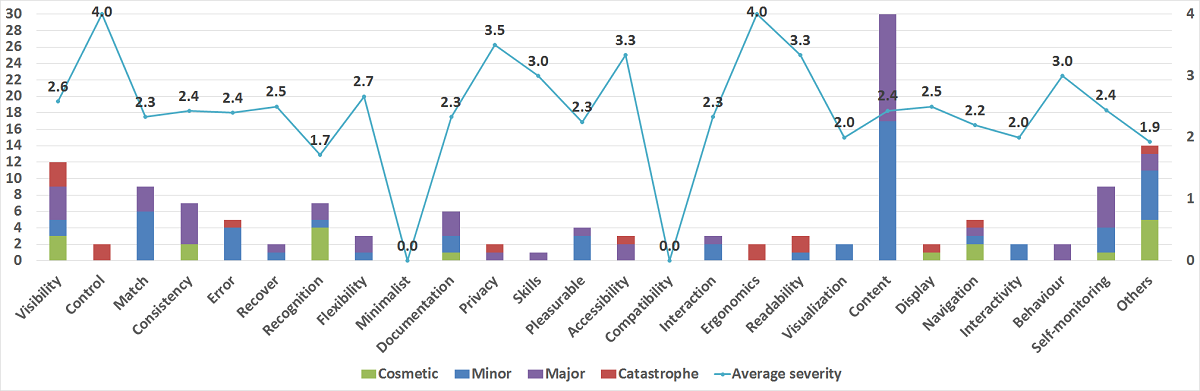
Usability problems identified using a proposed set of heuristics.

The results of the heuristic evaluation study are shown in Figure 4. The stacked columns show the number of usability problems found for each heuristic under zero, one, or more severity levels, while the line with markers shows the average severity rating of all the usability problems found for each heuristic. The horizontal axis shows the 25 heuristics of the set, and there are 2 vertical axes. The vertical axis on the left is the primary axis and represents the number of usability problems found, while the vertical axis on the right side shows the severity rating.

**Table 2:** Details of the participants in the expert evaluation study 2.

Parameter		Results, n (%)
**Gender**		
	Male	2 (29)
	Female	5 (71)
**Age group (years)**		
	21-25	3 (43)
	26-30	1 (14)
	31-35	1 (14)
	36-40	2 (29)
**Highest degree**		
	Bachelor’s degree	1 (14)
	Master’s degree	4 (57)
	Doctorate	2 (29)
**Employment status**		
	Full-time student	5 (71)
	Full-time employment and part-time student	2 (29)
HCI^a^ course^b^		7 (100)
**Previous app evaluation using a set of heuristics or guidelines**		
	1-4	3 (43)
	≥5	4 (57)

^a^HCI: human-computer interaction.

^b^Courses included HCI, interactive design, health care interaction design, and information visualization.

The cumulative number of usability problems found by all the groups was 137: severity rating 4: 15/137, 11.0%; severity rating 3: 49/137, 35.8%; severity rating 2: 54/137, 39.4%; severity rating 1: 19/137, 13.9%.

#### Usability Problems

The results show that one or more usability problems were found for most of the heuristics (23/25, 92%). The most frequently broken heuristics included content (30/137), visibility (12/137), and match and self-monitoring (9/137 each).

During the process of dividing the checklists into the groups, it was expected that some groups might find a usability problem in the app but that they may not find suitable checklist items to which they could map the problem. This was expected because each group received about 25% of the entire checklist. During the analysis of the usability problems, the groups found 14 usability problems for which they could not find a matching checklist item. In Figure 3, these usability problems are presented in the last stacked column labeled Others.

Multimedia Appendix 8 presents the details of some of the usability problems found by the participants. The details presented include heuristic number, problem description, the potential solution from their perspective, and severity rating. An “OT” in the first column of the table means the participants were unable to find a matching heuristic for the mentioned usability problems.

#### Average Severity Ratings

The average severity rating of all usability problems found was 2.4; this shows that most of the usability problems found were minor.

#### System Usability Scale (SUS)

Table 3 shows the SUS scores of each participant for the OneTouch app. The scores ranged from 42.5 through 75, with an average score of 60.

**Table 3:** System usability scale (SUS) scores.

Participant ID	SUS score
1	50.0
2	70.0
3	47.5
4	72.5
5	62.5
6	75.0
7	42.5

There are different ways to interpret the SUS scores [27]; these include percentiles, grades, adjectives, acceptability, and promoters and detractors. In this research, SUS scores were interpreted using acceptability, which defines the score in terms of what is “acceptable” or “not acceptable.”

According to the grading scale interpretation of SUS scores by Bangor and colleagues [28], the OneTouch app is Marginally acceptable. This shows that the OneTouch app needs enhancements to improve the usability of the app.

## Discussion

This research presents a modified set of heuristics called the HE4EH and its applicability by evaluating the OneTouch app. The HE4EH consists of Nilsen’s heuristic, mobile heuristics, health heuristics, and other heuristics (behavior change and self-monitoring of health). The HE4EH is a tool to assist HCI experts and mobile app developers when designing or evaluating an mHealth app for individuals with diabetes, by measuring the usability problems that can influence the user experience with the app. First, an expert evaluation study was conducted to improve heuristics and associated checklist items. Then, another expert evaluation study was conducted to evaluate a popular app called OneTouch intended to help with blood glucose management. The findings of the usability evaluation are as follows:

- Although the participants had a partial list of checklist items, they were able to identify the usability problems and link them to the given checklist items.
- The participants were also able to identify usability problems that could not be mapped to the partial list of given checklist items. This shows that, if given a complete list of checklist items, more usability problems could be identified.
- The top 3 frequently violated heuristics included Content, followed by Visibility, Match, and Self-monitoring.
- Although minimal, the participants were able to identify usability problems in the OneTouch app associated with the heuristics incorporated explicitly for behavior change and self-monitoring of blood glucose. The lack of incorporation of these and related heuristics and the associated checklist items means the identified usability problems had remained undetected.
- The average severity rating of all the usability problems found was minor.
- The mean SUS score showed that the OneTouch app is marginally acceptable and needs enhancements to improve the overall usability of the app.

### Limitations

As with all studies, this research has a few limitations. First, we used only selected bibliographic databases and search terms to identify a set of related heuristics. Second, a limited set of checklist items was given to each expert in the study. Last, the severity rating scale typically used in heuristic evaluation studies was also used in this research due to a subtle difference between the severity rating scales proposed in this research.

### Future Work

The current research can be extended in different ways. First, we intend to evaluate the mHealth apps by giving a complete set of checklist items to the HCI experts and investigate the impact on identifying usability problems in the apps, especially in terms of the specialized heuristics added to the HE4EH. They can consider using one of the severity rating scales discussed in this research. Second, future research can evaluate the effectiveness of different severity ratings proposed in this research. Third, the accessibility heuristic contains one generic checklist item; future research can, therefore, enrich it with a specialized set of guidelines like the Web Content Accessibility Guidelines [29] and then investigate the accessibility of mHealth apps. Similarly, checklist items for heuristics can also be adapted (eg, for privacy [7]). Fourth, future research can adapt the HE4EH and incorporate specific types of heuristics with more checklist items not covered in the set, especially in terms of controlling blood glucose monitoring. Fifth, future research can also adapt the HE4EH for behavioral change and self-monitoring of other health-related issues such as obesity and depression, among others. Last, future research can also conduct a qualitative study with domain experts to identify the heuritics or checklist items that can be combined to reduce the number of heuristics or checklist items.

## Appendix

Multimedia Appendix 1 Set of heuristics and checklist items.

Multimedia Appendix 2 Responses to the experts' satisfaction questionnaire.

Multimedia Appendix 3 Expert opinion on the heuristics.

Multimedia Appendix 4 Mean score of the checklist items, as scored by the experts.

Multimedia Appendix 5 Mean scores for severity rating scale 1.

Multimedia Appendix 6 Mean scores for severity rating scale 2.

Multimedia Appendix 7 Mean scores for severity rating scale 3.

Multimedia Appendix 8 Participants’ comments about issues with the heuristics.

## Abbreviations

HCI: human-computer interaction
HE4EH: heuristic evaluation for mHealth apps
mHealth: mobile health
SUS: system usability scale
