# Supplementary material for: New Checklist for the Heuristic Evaluation of mHealth Apps (HE4EH): Development and Usability Study
Source: JMIR Mhealth Uhealth. 2020 Oct 28;8(10):e20353. doi: 10.2196/20353 (PMC7657716; doi:10.2196/20353)
Supplement: Multimedia Appendix 1 [file mhealth_v8i10e20353_app1.docx]

## Heuristic #1: Visibility of system status.

1. Is there some form of system feedback for every operator action?
2. If pop-up windows are used to display error messages, do they allow the user to see the field in error?
3. In multipage data entry screens, is each page labeled to show its relation to others?
4. Are high informative contents placed in high hierarchy areas?
5. Do all the items on a list are on the same page? Are they sorted in an order that matches the needs of the task?
6. If a list of items can be sorted according to different criteria, does it provide an option to sort them using those criteria?
7. If a list contains items that belong to different categories, are there filters for users to narrow down the number of elements that they need to inspect?
8. If the list contains only one item, is the user taken directly to that item?
9. If the list contains items that download slowly (e.g., images), is the list split into multiple pages to show just one page at a time?
10. For articles that span several pages, is pagination shown at the bottom? Is there a link to each individual page, rather than just to the previous and the next ones?
11. Is the logo meaningful, identifiable, and sufficiently visible?
12. Is there any link to detailed information about the enterprise, web site, webmaster, etc.?
13. Are there ways of contacting with the enterprise?
14. In articles, news, reports, etc. are the author, sources, dates, and review information shown clearly?
15. For physical location information on the website, is a link to a map provided and the directions clearly accessible?
16. Are response times appropriate for the user's cognitive processing?
17. Are response times appropriate for the task?
18. If there are observable delays (greater than fifteen seconds) in the system’s response time, is the user kept informed of the system progress?
19. Is latency sufficiently reduced?
20. Are splash screens too long avoided?
21. If download time is greater than 20 seconds, are progress bars offered instead of non-informative download screens?
22. Is there visual feedback in menus or dialogue boxes about which choices are selectable?
23. Is the current status of an icon clearly indicated?
24. Is there visual feedback when objects are selected or moved?
25. Are links recognizable? Is there any characterization according to the state (visited, active, etc.)?
26. Are low discoverable areas as touch buttons well identifiable?
27. When swiping gesture is possible, is a visible clue offered to users? Is swiping used with a unique meaning in the same screen?
28. Are expandable menus used sparingly? Do menu labels clearly indicate that they expand to a set of options?
29. When users access the site from a mobile phone, are they directed to the mobile version of the site?
30. Is a link to the mobile site provided on the desktop version of the site?
31. Is a link to the full site included on the mobile page?
32. For interface design in mobile phones, Is clear and concise information about the status of the system provided on every screen?
33. Is critical and contextual information, such as battery status, network status, environmental conditions, etc. prioritized?
34. Is sound and light feedback explored as an alternate of text feedback? However, such resources must be used with discretion to avoid disturbing the user (Alsos et al., 2012).
35. Is the device keep the user informed about all processes and state changes through comments within a reasonable time frame?

## Heuristic #2: User control and freedom

1. Users often choose system functions by mistake, is there any option marked with “emergency exit” for the user to leave the unwanted state without having to go through an extended dialogue?
2. Are undo and redo of actions performed by the user supported by the system?
3. Can users move forward and backward between fields or dialogue box options?
4. If the system has multipage data entry screens, can users move backward and forward among all the pages in the set?
5. If the system uses a question and answer interface, can users go back to previous questions or skip forward to later questions?
6. Are exits clearly marked?
7. Is the general web site structure user-oriented?
8. Is there any way to inform the user about where they are and how to undo their navigation?
9. Is accidental activation avoided or foreseen (a back button is offered)?
10. In mobile websites, is navigation on the homepage provided?
11. Can users set their own system, session, file, and screen defaults?
12. When a user's task is complete, does the system wait for a signal from the user before processing?
13. Are users prompted to confirm commands that have drastic, destructive consequences?
14. Can users easily reverse their actions?
15. Can users cancel out of operations in progress?
16. If the system has multiple menu levels, is there a mechanism that allows users to go back to previous menus?
17. Are menus broad (many items on a menu) rather than deep (many menu levels)?
18. If users can go back to a previous menu, can they change their earlier menu choice?
19. Are interruptions made by the user treated naturally by the system?
20. Is the user provided with a possibility to immediately restart his/her actions after interruptions?
21. Are controls of basic navigation provided on the screen even if the device itself provides buttons to perform these functions?
22. Are horizontal and/or vertical navigation options offered by the system should be intuitive?
23. Are these options (undo, redo, exits) available through a physical button or equivalent?

## Heuristic #3: Match between system and real world

1. Is the system speaking the users’ language, with words, phrases, and concepts familiar to the user, rather than system-oriented terms?
2. Is real-world convention followed in the system?
3. Is information displayed in a logical and natural order?
4. Are metaphors properly used as visual clues?
5. Are icons concrete and familiar?
6. If the shape is used as a visual cue, does it match cultural conventions?
7. Do the selected colors correspond to common expectations about color codes?
8. If the site uses a hierarchical structure, are depth and height-balanced?
9. Is a navigation map or table of contents included on the site?
10. Is too much navigation avoided?
11. Are menu choices ordered in the most logical way, given the user, the item names, and the task variables?
12. Do menu choices fit logically into categories that have readily understood meanings?
13. Are menu titles parallel grammatically?
14. In navigation menus, are the number of items and terms by item controlled to avoid memory overload?
15. Do related and interdependent fields appear on the same screen?
16. For question and answer interfaces, are questions stated in clear, simple language?
17. Is the language used the same target users speak?
18. Is the language clear and concise?
19. Does the site follow the rule "1 paragraph=1 idea"?
20. Does the system automatically enter leading or trailing spaces to align decimal points?
21. Does the system automatically enter a dollar sign and decimal for monetary entries?
22. Does the system automatically enter commas in numeric values greater than 9999?
23. Are integers right-justified and real numbers decimal-aligned?
24. The most natural and easy to use operation modes are obtained by making the same experience from daily life events, is the natural mode extended to smartphones?
25. Are gestures such as glide, drag, rotation applied in the daily life appear/look natural?
26. Does the app take reliance on user models that come from prior technologies?

## Heuristic #4: Consistency and standards

1. Does the app let users wonder whether different words, situations, or actions mean the same thing?
2. Are platform conventions followed?
3. Is constraining orientation avoided? (Users tend to switch orientation when an impasse occurs and, if the app doesn’t support them, their flow is going to be disrupted, and they are going to wonder why it’s not working)
4. Is navigation (horizontal and vertical) consistent across orientations? (Some applications use a different navigation direction in the two orientations; for instance, they use horizontal navigation in landscape and use vertical navigation in portrait).
5. Is content consistent across orientations?
6. Are attention-getting techniques used with care?
7. Is intensity maintained in two levels only?
8. Is the number of colors used constrained up to four? Are additional colors saved for occasional use only?
9. Are the colors far apart along the visible spectrum?
10. Are soft tones used for the regular positive feedback and harsh for rare critical conditions?
11. If the system has multipage data entry screens, do all pages have the same title?
12. Do on-line instructions appear in a consistent location across screens?
13. Have industry or company standards been established for menu design, and are they applied consistently on all menu screens in the system?
14. Are there no more than twelve to twenty icon types?
15. Has a heavy use of all uppercase letters on a screen been avoided?
16. Is there a consistent icon design scheme and stylistic treatment across the system?
17. Are menu choice lists presented vertically?
18. If "exit" is a menu choice, does it always appear at the bottom of the list?
19. Are menu titles either centered or left-justified?
20. Are field labels consistent from one data entry screen to another?
21. Do field labels appear to the left of single fields and above list fields?
22. Are field labels and fields distinguished typographically?
23. Is the structure of a data entry value consistent from screen to screen?
24. Are system objects named consistently across all prompts in the system?
25. Are user actions named consistently across all prompts in the system?
26. Are names of menu choices consistent, both within each menu and across the system, in grammatical style and terminology?
27. Does the structure of menu choice names match their corresponding menu titles?
28. Does the menu structure match the task structure?
29. When prompts imply a necessary action, are the words in the message consistent with that action?
30. Where are the web site goals? Are they well defined? Do content and services delivered match these goals?
31. Does the look & feel correspond with goals, characteristics, contents, and services of the web site?
32. Is the web site updated frequently?
33. Is system response after clicking links predictable?
34. Are nowhere links avoided?
35. Are orphan pages avoided?
36. Is mobile interface similar to the desktop interface? In terms of buttons, logos, and color schemes so that user experience is consistent across all platforms?
37. Are link labels emphasized in graphic elements?
38. Does the app maintain the components in the same place and look throughout the interaction?
39. Are similar functionalities performed by similar interactions?
40. Does the metaphor of each component or feature unique throughout the application, to avoid misunderstandings?

## Heuristic #5: Error prevention

1. Even better than good error messages is a careful design which prevents a problem from occurring in the first place. Does application either eliminate error-prone conditions or check for them and present users with a confirmation option before they commit to the action?
2. Are menu choices logical, distinctive, and mutually exclusive?
3. Are data inputs case-blind whenever possible?
4. Does the system warn users if they are about to make a potentially serious error?
5. Do data entry screens and dialogue boxes indicate the number of character spaces available in a field?
6. Do fields in data entry screens and dialogue boxes contain default values when appropriate?
7. Is accidental activation avoided or foreseen (a back button is offered)?
8. Are touchable areas sufficiently big (Research has shown that the best target size for widgets is 1cmx1cm for touch devices)?
9. Is crowding targets avoided (When targets are placed too close to each other, users can easily hit the wrong one)?
10. Although the visible part of the target may be small, is there some invisible target space that if a user hits that space, their tap will still count?
11. When several items are listed in columns, one on top of another, can users hit anywhere in the row to select the target corresponding to that row?
12. Is downloading software that is inappropriate for the user phone avoided?
13. Are JavaScript and Flash use avoided?
14. Is confirmation from the user taken before performing, running, or using complex procedures to avoid accidental errors?
15. Are unavailable features hidden or disabled?
16. Are users warned about the critical actions and access provided to additional information?

## Heuristic #6: Help users recognize, diagnose, and recover from errors

1. Are error messages expressed in plain language (no codes) familiar language of the user?
2. Does the error message precisely indicate the problem and constructively suggest a solution?
3. When signaling an input error in a form, is the text box that needs to be changed specifically marked or text highlighted in it?
4. Due to limited data entry and, consequently, higher rates of user errors, do mobile phones provide meaningful feedback? Concise error message? Return options and easy error recovery?

## Heuristic #7: Recognition rather than recall

1. Are instructions to use system visible or easily retrievable whenever appropriate?
2. Are high levels of concentration not required, and remembering information doesn't take more than two to fifteen seconds?
3. Are all data a user needs on display at each step in a transaction sequence?
4. If users have to navigate between multiple screens, does the system use context labels, menu maps, and place markers as navigational aids?
5. After the user completes an action (or group of actions), does the feedback indicate that the next group of actions can be started?
6. Are optional data entry fields clearly marked?
7. Do data entry screens and dialogue boxes indicate when fields are optional?
8. Is page length controlled?
9. Should the task flow start with actions that are essential to the main task? And can the users start the task as soon as possible?
10. Are the controls that are related to a task grouped together and reflect the sequence of actions in the task?
11. For question and answer interfaces, are visual cues and white space used to distinguish questions, prompts, instructions, and user input?
12. Does the data display start in the upper-left corner of the screen?
13. Have prompts been formatted using white space, justification, and visual cues for easy scanning?
14. Do text areas have "breathing space" around them?
15. Are there "white" areas between informational objects for visual relaxation?
16. Does the system provide visibility: that is, by looking, can the user tell the state of the system and the alternatives for action?
17. Are size, boldface, underlining, color, shading, or typography used to show relative quantity or importance of different screen items?
18. Is color used in conjunction with some other redundant cue?
19. Is there good color and brightness contrast between image and background colors?
20. Have light, bright, saturated colors been used to emphasize data and have darker, duller, and desaturated colors been used to de-emphasize data?
21. Is the visual page space well used?
22. On data entry screens and dialogue boxes, are dependent fields displayed only when necessary?
23. Are field labels close to fields, but separated by at least one space?
24. Is the first word of each menu choice the most important?
25. Are inactive menu items greyed out or omitted?
26. Are there menu selection defaults?
27. Is there an obvious visual distinction made between "choose one" menu and "choose many" menus?
28. Is there a breadcrumb on sites with a deep navigation structure (many navigation branches)? And, is it avoided on sites with shallow navigation structures?
29. Does the device provide visible objects, actions, and options to prevent users from having to memorize information from one part of the dialogue box to another?
30. Are objects, actions, and options visible to the user to minimize its memory load?

## Heuristic #8: Flexibility and efficiency of use

1. Accelerators are typically unseen by the novice user but can often speed up the interaction for the expert user. Are accelerators used?
2. Is the searching box easily accessible?
3. Is the searching box easily recognizable?
4. Is there any advanced search option?
5. Are search results shown in a comprehensive manner to the user?
6. Is the box width appropriated?
7. Is the user-assisted if the search results are impossible to calculate?
8. Is there a search box on the mobile site homepage?
9. Is the length of the search box at least the size of the average search string? Or better, is it the largest possible size that will fit on the screen?
10. Are search strings preserved between searches? Are there auto-completion and suggestions?
11. Are several search boxes with different functionalities on the same page avoided?
12. If the search returns zero results, are some alternative searches offered or a link to the search results on the full page?
13. Are links with good information scent (that is, links which clearly indicate where they take the users)?
14. Are there links to related content to help the user navigate more quickly between similar topics?
15. Does the device charge fast?
16. Is the device ease to learn?
17. To increase interaction efficiency, does the device provide an alternative way to display the same function as a shortcut?
18. Are all features found in a single interaction?
19. Whenever possible, does the system suggest support and provide customization options for frequent actions?
20. Does the device provide the facility of basic and advanced settings to set and customize the shortcuts for frequent actions?
21. Is information loaded and displayed in a reasonable amount of time?
22. Are a number of steps required to perform a task minimally?
23. Are animations and transitions displayed seamlessly?

## Heuristic #9: Aesthetic and minimalist design

1. Every extra unit of information in a dialogue competes with the relevant units of information and diminishes their relative visibility. Do dialogues contain information that is relevant and frequently needed?
2. Is the time to acquire a target is a function of the distance to and size of the target following Fitts Law?
3. Is only (and all) information essential to decision making displayed on the screen?
4. Are field labels brief, familiar, and descriptive?
5. Are prompts expressed in the affirmative, and do they use the active voice?
6. Is the layout clearly designed to avoid visual noise?
7. Are application icons recognizable enough to be found in the crowded list of applications?
8. Does the use of images and multimedia content add value?
9. Are images well-sized? Are they understandable? Is the resolution appropriate?
10. Are cyclical animations avoided?
11. Is flash content avoided?
12. Is the use of animated carousels avoided? And if they exist, can users control them?
13. Are image sizes smaller than the screen (The entire image should be viewable with no scrolling)?
14. For cases where customers are likely to need access to a higher resolution picture, is a screen-size picture initially displayed, and is there a separate link to a higher resolution variant?
15. When using thumbnails, can the user distinguish what the picture is about?
16. Do captions help to understand images meaning that are part of an article if their meaning is not clear from the context of the article?
17. Are moving animation avoided?
18. When using videos, is there a textual description of what the video is about?
19. Do clicking on the thumbnail and clicking on the video title both play the video?
20. Is the video length indicated?
21. If the video cannot be played on the user’s device, is it a message shown with this information?
22. Is the whole screen surface used to place information efficiently (especially for popovers and modals)?
23. Has excessive detail in icon design been avoided?
24. Is each individual icon a harmonious member of a family of icons?
25. Does each icon stand out from its background?
26. Are all icons in a set visually and conceptually distinct?
27. Is each lower-level menu choice associated with only one higher-level menu?
28. Are menu titles brief, yet long enough to communicate?
29. Desktop websites have a strong guideline to avoid horizontal scrolling. But for touch screens, horizontal swipes are often fine. Is this option taken into account?
30. Is the site designed to avoid a large number of persistent navigation options across all pages?
31. Is the information presented consistently and divided into small portions? And easy to find to minimize the cognitive load of the user?
32. To access the complete version of the application, does the mobile phone allow the user to access the desktop versions of the site from their mobile phone?
33. Is the screen area partially used by a keyboard?
34. The most relevant information should be highlighted visually through a larger size, color, use of markers, etc. Is information highlighted that way visually?
35. Due to the size limit of the screen, unlike websites for desktop, the navigation is NOT repeated on all the pages?
36. Is the display of unwanted information avoided to overload the screen?

## Heuristic #10: Help and documentation

1. Even though it is necessary to provide help and documentation as a part of the system, but is the system in a condition that it can be used without the support of documentation?
2. Is the information in the help and documentation easy to search, focused on the user’s task, list concrete steps to be carried out, and not be too large?
3. Are on-line instructions visually distinct?
4. Do the instructions follow the sequence of user actions?
5. If menu choices are ambiguous, does the system provide additional explanatory information when an item is selected?
6. If menu items are ambiguous, does the system provide additional explanatory information when an item is selected?
7. Is the help function visible, for example, a key labeled HELP or a special menu?
8. Is the help system interface (navigation, presentation, and conversation) consistent with the navigation, presentation, and conversation interfaces of the application it supports?
9. Is the information easy to find?
10. Is the visual layout well designed?
11. Is the information accurate, complete, and understandable?
12. Is the information relevant? It should be relevant in the following aspects: Goal-oriented (What can I do with this program?), Descriptive (What is this thing for?), Procedural (How do I do this task?), Interpretive (Why did that happen?) and Navigational (Where am I?).
13. Is there context-sensitive help?
14. Can the user change the level of detail available?
15. Can users easily switch between help and their work?
16. Is it easy to access and return from the help system?
17. Can users resume work where they left off after accessing help?
18. If a FAQs section exists, is the selection and redaction of questions and answers correct?
19. Is the design focused on one single feature at a time? (Only those instructions that are necessary for the user to get started should be presented at a time).
20. Does the application provide a help option where common problems and ways to solve them are specified?
21. Are issues considered in the help option easy to find?
22. Does the device provide documentation that is easy to find and help, focusing on the user’s current task and indicating concrete steps to follow?

## Heuristic #11: Privacy

1. Are protected areas completely inaccessible?
2. Are protected or confidential areas only accessible with certain passwords?
3. Is there information about how personal data is protected and about contents copyright?
4. For multiuser devices: Is permanently signing in on an app avoided?
5. If the app does store credit card info, can users decide if they want to remain logged in? If the user opts to be kept logged in, he/she should get a message informing of the possible risks.
6. Is the user’s confidential data protected by the device?

## Heuristic #12: Skills

1. Is the word "default" avoided and replaced with "Standard," "Use Customary Settings," "Restore Initial Settings," or some other more specific terms describing what will actually happen?
2. If the system supports both novice and expert users, are multiple levels of error message detail available?
3. If the system supports both novice and expert users, are multiple levels of detail available?
4. Are users the initiators of actions rather than the responders?
5. Do the selected input device(s) match user capabilities?
6. Are important keys (for example, ENTER, TAB) larger than other keys?
7. Does the system correctly anticipate and prompt for the user's probable next activity?

## Heuristic #13: Pleasurable interaction

1. Is the users' work protected? For example, for data entry screens with many fields or in which source documents may be incomplete, can users save a partially filled screen?
2. Do the selected input device(s) match environmental constraints?
3. Are typing requirements minimal for question and answer interfaces?
4. Does the system complete, unambiguous partial input on a data entry field?
5. Users dislike typing. Is information computed for the users? For instance, ask only for the zip code and calculate state and town; possibly offer a list of towns if there are more under the same zip code.
6. Is the input data tolerant of typos and offers corrections? (Don't make users type incomplete information. For example, accept “123 Main” instead of “123 Main St.”)
7. Can users save history and select previously typed info?
8. Does default information make sense to the user?
9. If the app does not store any information that is sensitive (e.g., credit card), is the user kept logged in (with the log out clearly presented)?
10. Is the number of submissions (and clicks) minimized for the user going through in order to input information on the site?
11. When logging in must be done, are graphical passwords used at least some of the time, to get around typing?
12. Is registration not mandatory? Is skipping registration the default option?
13. When logging in must be done, is there an option that allows the user to see the password clearly?
14. When a list of products is presented, are image thumbnails big enough for the user to get some information out of them?
15. On a product page, does image size fit the screen? Is there a link to a higher resolution image when the product requires closer inspection?
16. Is there the option to email a product to a friend?
17. Is there the option to save the product in a wish list?
18. On an e-commerce site, are salient links included on the homepage to the following information: — locations and opening hours (if applicable), — shipping cost, — phone number, — order status, and — occasion-based promotions or products?
19. Whenever users conduct transactions on the phone, can they save confirmation numbers for that transaction by emailing themselves? If the phone has an embedded screen-capture feature, are there instructions about how to take a picture of their screen?
20. Does the device provide a pleasant iteration with the user so that the user does not feel uncomfortable while using the application?

## Heuristic #14: Accessibility

1. Is the app accessible to people with disabilities?

## Heuristic #15: Compatibility between different platforms

1. Considering the large fragmentation and constant innovation of the mobile device market, is application flexible to adapt to different platforms and devices?
2. Does the site or application automatically detect the kind of device and direct the user to the appropriate mobile version?

## Heuristic #16: Minimized human/device interaction

1. Considering the size of the mobile device and its context of use, is the interaction effort reduced? For instance, by placing the search box and navigation controls on the homepage.
2. Is scrolling avoided, especially to view the URLs?
3. Is scrolling hidden by default? And becomes visible only when there is an interaction between the user and the device?
4. Is data entry by the user reduced to minimal by providing them with a number of features like auto-complete, text-boxes with suggestions, buttons, menus, lists of predefined values?
5. Is historical and personalization data retained to establish default values, such as a cache of frequent responses?
6. Are typos and abbreviations tolerated?
7. Are registration and login avoided?

## Heuristic #17: Physical interaction and ergonomics

1. Are the most common navigation controls within reach of the user?
2. Can most common navigation controls be easily pressed by the thumb of the user?
3. Do touch-sensitive control elements have adequate size and spacing that users can easily touch them with their fingers?
4. Is text representing links using about or under three words?
5. Is the contact zone of the touch control has the same size of the icon displayed on the screen?

## Heuristic #18: Readability and layout

1. Do the text boxes fit on the screen of a mobile device?
2. Can screen layout fit multiple devices of different screen sizes like (176x220, 128x160, 320x480)?
3. Is the content of the screen easy to read in different lighting conditions?
4. Is there sufficient contrast between text and background, icons, and background? Especially in the case of backgrounds with images?
5. Are a limited number of colors used?
6. Is textual description as an alternative to information displayed with graphics provided?
7. Is the content readable in both landscape and portrait?

## Heuristic #19: Non-interruptive app information visualization

1. Are mobile applications NOT interrupting the current activity of the user?
2. Are additional messages from the applications running in the background NOT occupying the user’s peripheral vision?
3. To know if the user can be interrupted, is a system able to perceive the environment and adapt the presentation of information accordingly?
4. Is alternative feedback method sound, light, flash, graphic information (e.g., static/animated icons, or text notifications) or vibration used to avoid distracting the user from the primary tasks?

## Heuristic #20: Content

1. Is the home or main screen simple and engaging?
2. Are registration and logging screens simple and obvious as possible?
3. Is the most important information put in the hierarchy first?
4. Is the user informed about what to do and how to do it?
5. The user needs to stay positive and realistic, are the benefits of taking actions included?
6. Are specific steps to perform each action provided/described?
7. Is the content written in plain language?
8. Is the accuracy of the content checked?
9. Is content clearly displayed on each screen?
10. Is the amount of interactive content that users can tailor limited?
11. Are headings meaningful?

## Heuristic #21: Display

1. Are styles consistent?
2. Are fonts easy to read?
3. Are white spaces used to avoid clutter?
4. Is content placed in the center of the screen and above the fold?
5. The images used across the screen are easy to learn and remember?
6. Are colors bold with contrast? And are dark and busy backgrounds avoided?

## Heuristic #22: Navigation

1. Are the topics placed in multiple categories?
2. Are home and menu screens easy to access?
3. Is the “Back” button working properly?
4. Is information placed in a linear order (e.g., numbered screens)?
5. Are screen-based controls easy to navigate?
6. Is the size of buttons large enough to use?
7. Are links clearly labeled to ensure their effective use?
8. Are options of search and browse provided?

## Heuristic #23: Interactivity

1. Are users invited to share content and provide feedback about their experiences?
2. Are printer-friendly tools and resources included?
3. Are audio and visual features incorporated?
4. Are new media such as Twitter and text messaging explored?

## Heuristic #24: Behavior change

1. Is feedback on performance was given by providing data about recorded behavior or evaluating performance in relation to a set standard or others’ performance.
2. Is social support or social change provided by prompting consideration of how others’ could change their behavior to offer the person help or (instrumental) social support, including “buddy” systems – and/or providing social support?
3. Is information about others’ approval provided, e.g., what others think about the person’s behavior and whether others will approve or disapprove of any proposed behavior change?
4. Are contingent rewards in terms of praise, encouragement, or material rewards given that are explicitly linked to the achievement of specified behaviors?
5. Is the user-provided general encouragement by praising or rewarding the person for effort or performance without this being contingent on specified behaviors or standards of performance?
6. Are instructions provided in the app by telling the person how to perform a behavior and/ or preparatory behaviors?
7. Is the user provided with general information about behavioral risk, e.g., susceptibility to poor health outcomes or mortality risk in relation to the behavior?
8. Are opportunities for social comparison provided by facilitating observation of non-expert others’ performance, e.g., in a group class or using video or case study?
9. Is functionality to model/demonstrate the behavior of an expert provided in an app that shows the person how to correctly perform a behavior, e.g., in class or on video?
10. Is the setting of graded tasks supported by providing a facility to set easy tasks, and increasing difficulty until target behavior is performed?
11. Is the functionality of time management provided by helping the person make time for the behavior (e.g., to fit it into a daily schedule)?
12. Does the app provide an agreement (e.g., signing) of a contract specifying behavior to be performed so that there is a written record of the person’s resolution witnessed by another?
13. Is motivational interviewing supported in-app by prompting the person to provide self-motivating statements and evaluations of their own behavior to minimize resistance to change?
14. Does the app support stress management, which may involve a variety of specific techniques (e.g., progressive relaxation) that do not target the behavior but seek to reduce anxiety and stress?
15. Is relapse prevention supported by following initial change, help identify situations likely to result in re-adopting risk behaviors or failure to maintain new behaviors, and help the person plan to avoid or manage these situations?
16. Is functionality to teach the person to identify environmental cues provided, which can be used to remind them to perform a behavior, including times of day, contexts, or elements of contexts?
17. Does the app use follow-up prompts by contacting the person again after the main part of the intervention is complete?
18. Is functionality in which the user is asked to keep a record of specified behavior/s (e.g., in a diary) supported?
19. Is functionality to timely prompt specific goal setting that involves detailed planning of what the person will do, including a definition of the behavior specifying frequency, intensity, or duration, as well as the specification of at least one context, i.e., where, when, how or with whom supported?
20. Is the user provided with practice by prompting the person to rehearse and repeat the behavior or preparatory behaviors?
21. Does the app provide intention formation by encouraging the person to decide to act or set a general goal, e.g., to make a behavioral resolution such as “I will take more exercise next week”?
22. Is a review of behavioral goals and/or reconsideration of previously set goals or intentions provided in the app?
23. Is self-talk function that encourages the use of self-instruction and self-encouragement (aloud or silently) to support activities provided in the app?
24. Does the app prompt identification of itself as a role model for others by indicating how the person may be an example to others and influencing their behavior or providing an opportunity for the person to set a good example?
25. Does the app prompt for barrier identification, which resists to performing the behavior and plan ways of overcoming them?
26. Is the information on consequences provided in the app, e.g., the benefits and costs of action or inaction, focusing on what will happen if the person does/does not perform the behavior?

## Heuristic #25: Self-monitoring

1. Are self-monitoring of blood glucose (SMBG) protocols (intensity and frequency) incorporated in-app individualized to address each individual’s specific educational/behavioral/clinical requirements (to identify/prevent/manage acute hyper- and hypoglycemia) and provider requirements for data on glycaemic patterns and to monitor the impact of therapeutic decision making?
2. Does the app take an agreement or consent from the user with diabetes for the purpose/goal of performing SMBG and using SMBG data? And are these agreed/consented upon purposes/goals and actual review of SMBG data documented?
3. Is the SMBG in-app requires an easy procedure for patients to regularly monitor the performance and accuracy of their glucose meter?
4. Is the user informed that meter and test strips should be handled with clean, dry hands?
5. Does the user know through an app that test strips are for single-use and unique for each meter?
6. Is the user informed that test strips must be kept in the original canister, as any moisture can affect the integrity of the strip? And the containers should be kept closed?
7. Does the app inform the user to check for the expiration date?
8. Is the user informed that strips can be tested for accuracy with the control solution provided initially with each meter and should be checked for expiration date?
9. Does the user know through an app that control glucose range for the strips appears on the canister?
10. Does the app inform the user that some meters require coding with each canister? And many of the newer meters do not require coding.
11. Is the user informed that the amount of blood required is usually very small? Many meters easily pull the blood drop into the end of the strip. The inadequate sample can be a source of error.
12. Does the user know through the app to keep meter and supplies in a cool, dry area, not in the car or in sunlight?
13. Does the app inform the user to bring meter for office visits with a diabetes educator or primary care provider to test the accuracy comparatively?
14. Is the user informed to clean the area with warm, soapy water and dry? Food residue can be a source of false high blood sugar values.
15. Is the user informed to lancet devices to obtain blood can vary and all use a lancet to prick the skin? Thin, sharp lancets are more comfortable, and they should not be reused or cleaned, as they quickly become dull.
16. Is the user informed that the depth setting on the lancet device controls the penetration of the stick and can be adjusted for the best comfort and size of the blood sample? Most meters require very small samples—less than a small teardrop.
17. Is the user informed that lancet should be applied firmly to the clean, dry finger, but not with force?
18. Is the user informed that the sides of the finger should be used, as there is less pain? Use of the third, fourth, and fifth digits may be preferable to spare index finger and thumb.
19. Is the user informed that alternate test sites (upper arms and thighs) are approved for many meters? Fingertips or the outer palm are preferred and are more accurate.
20. Is the user informed that the obtainment of the blood sample should be a gentle “milking” from the base of the finger to the lanced tip? Pressure directly on the site of lancing is not recommended.
21. Is the user informed that disposal of lancets and SMBG testing supplies should be done according to local laws for sharps? In many locations, a hard plastic container with a screw-top can be disposed of in the household trash.
22. Is the app using simple and specific steps at the patient’s level of comprehension?
23. Is the user informed through an app to ask a diabetes educator or primary care provider for frequency and times of testing and desired results?
24. Does the app remind the user to observe the SMBG procedure at follow-up visits?
25. Does the user know through the app to ask the patient to assess the relationship of SMBG with exercise, food, medications, and stress?
26. Does the app acknowledge the patient for goals achieved with SMBG?
27. Is the user informed that using blood glucose monitoring technology requires the use of glucose strips?
28. Blood glucose monitoring technology is the common form of glucose monitoring, does app inform the user about this?
29. Does the app inform the user that the frequency of glucose readings depends upon how often the person pricks their finger?
30. Does the user know through an app that blood glucose monitoring technology doesn’t show glucose trends? Some meters can show patterns if glucose checks are done at the same time over several days.
31. Memory in blood glucose monitoring technology varies depending on the meter chosen, is user informed through an app?
32. Does the user know that time taken for glucose check using blood glucose monitoring technology is typically 1-2 minutes as it varies depending on the meter?
33. Is the user informed that there is no alarm in blood glucose monitoring technology, but alerts (no alarms) can be shown on smart devices for low and high blood glucose levels?
34. Does the app inform the user that for blood glucose monitoring technology, tips, or sides of fingers can be used for sampling?
35. Some meters need intermittent calibration using a solution in blood glucose monitoring technology, is this informed to the user?
36. Is the user informed that the sensor in flash glucose monitoring technology works for 14 days?
37. Does the user know through an app that flash glucose monitoring technology shows glucose trends?
38. Does the app inform the user that the frequency of glucose readings depends on how often the sensor is scanned? The device automatically samples interstitial glucose every 15 minutes and stores in memory for 8 hours.
39. Does the user know through an app that flash glucose monitoring technology shows glucose trends when the sensor is scanned, the transmitter displays the past 8 hours of glucose levels?
40. The memory of the device used in flash glucose monitoring technology stores reading from the past 8 hours, is user informed through an app?
41. Does the user know that time taken for glucose check-in flash glucose monitoring technology includes 1 second of scan and Inserting sensor takes a few minutes?
42. Is the user informed that there is no alarm in flash glucose monitoring technology, but alerts (no alarms) can be shown on the device, but only if the scan is done at a time when glucose is low or high?
43. Does the app inform the user that flash glucose monitoring technology is approved for use on the upper arm to collect samples?
44. Calibration is not needed in flash glucose monitoring technology, is this informed to the user?
45. Is the user informed that the sensor in CGM technology works for 6 to 7 days?
46. Real-time glucose readings are taken using CGM technology, is user informed through an app?
47. Does the user know through an app that CGM technology shows glucose trends?
48. Is the user informed that CGM technology alarms when glucose levels are out of target range?
49. Does the app inform the user that the frequency of glucose readings in CGM is automatic and samples interstitial glucose every 5 minutes and shows readings on a graph?
50. Does the user know through an app that CGM technology shows glucose trends?
51. The memory of the device used in CGM technology displays and stores up to 24 hours of glucose readings, is user informed through an app?
52. Does the user know that time taken for glucose check using CGM technology is based on the data viewed on-demand on a smart device, receiver, or insulin pump?
53. Is the user informed that devices in CGM technology can be programmed to alarm for low and high glucose levels?
54. Does the app inform the user that CGM technology is approved for use on the stomach to collect samples?
55. Finger prick calibration needed at least twice daily in CGM technology, is this informed to the user?
56. Is the user informed that the sensor in CGM with an insulin pump technology works for 6 to 7 days?
57. Real-time glucose readings are taken using CGM with an insulin pump technology, is user informed through an app?
58. Does the user know through an app that CGM with an insulin pump technology shows glucose trends?
59. Is the user informed that CGM technology alarms when glucose levels are out of target range?
60. CGM technology is integrated with an insulin pump, is user informed through an app?
61. Does the app inform the user that the frequency of glucose readings in CGM automatically and samples interstitial glucose every 5 minutes and shows readings on a graph on the insulin pump?
62. Does the user know through an app that CGM with an insulin pump technology shows glucose trends?
63. The memory of the device used in CGM technology with an insulin pump displays and stores up to 24 hours of glucose readings, is user informed through an app?
64. Does the user know that time taken for glucose check using CGM with an insulin pump technology is based on the data viewed on-demand on an insulin pump?
65. Is the user informed that devices in CGM technology can be programmed to alarm for low and high glucose levels?
66. Does the app inform the user that CGM with an insulin pump technology is approved for use on the stomach to collect samples?
67. Finger prick calibration needed at least twice daily in CGM with an insulin pump technology, is this informed to the user?
