# Supplementary material for: New Checklist for the Heuristic Evaluation of mHealth Apps (HE4EH): Development and Usability Study
Source: JMIR Mhealth Uhealth. 2020 Oct 28;8(10):e20353. doi: 10.2196/20353 (PMC7657716; doi:10.2196/20353)
Supplement: Multimedia Appendix 2 [file mhealth_v8i10e20353_app2.docx]

Responses to the experts' satisfaction questionnaire.

| **Q#** | **Question** | **SA** | **AG** | **NE** | **DA** | **SD** |
| --- | --- | --- | --- | --- | --- | --- |
| 1 | I can evaluate a website/app feature quickly by applying the checklist items | 4 |  |  | 1 |  |
| 2 | I am satisfied with the number of checklist items included | 4 |  |  | 1 |  |
| 3 | The length of the HE4EH should be reduced | 3 |  | 1 | 1 |  |
| 4 | It is easy to understand how to apply the HE4EH on a website/app | 4 | 1 |  |  |  |
| 5 | The terminology used in the HE4EH is clear and easy to understand | 4 | 1 |  |  |  |
| 6 | I would need additional instructions to evaluate a website/app with HE4EH | 3 |  |  | 2 |  |
| 7 | It is easy to learn how to use the HE4EH once you have used it | 4 | 1 |  |  |  |
| 8 | Checklist items are well categorized under a heuristic | 4 |  |  |  | 1 |
| 9 | The amount of information included in the HE4EH was useful | 3 | 1 |  |  | 1 |
| 10 | The use of the HE4EH is not complex | 1 | 1 | 3 |  |  |
| 11 | The proposed HE4EH severity ratings are effective in measuring the degree of usability violations |  | 3 | 1 | 1 |  |
| 12 | The material used to develop the HE4EH is acceptable and sufficient? |  | 4 |  | 1 |  |
| 13 | Developing tools that improve the health of the patients and allow them to self-monitor and behavioral change. | 3 | 1 |  | 1 |  |

*Note. SA=Strongly Agree; AG=Agree; NE=Neutral; DA=Disagree; SD=Strongly Disagree.*
