# Supplementary material for: New Checklist for the Heuristic Evaluation of mHealth Apps (HE4EH): Development and Usability Study
Source: JMIR Mhealth Uhealth. 2020 Oct 28;8(10):e20353. doi: 10.2196/20353 (PMC7657716; doi:10.2196/20353)
Supplement: Multimedia Appendix 3 [file mhealth_v8i10e20353_app3.docx]

Expert opinion on the heuristics.

| **#** | **Heuristic** | **Importance** | | | **Relevance** | | | **Clarity** | | |
| --- | --- | --- | --- | --- | --- | --- | --- | --- | --- | --- |
|  |  | **VI** | **IM** | **MI** | **VR** | **RE** | **MR** | **VG** | **GD** | **BA** |
| 1 | Visibility of system status | 2 | 3 |  | 2 | 2 | 1 | 3 |  | 2 |
| 2 | User control and freedom | 4 | 1 |  | 3 | 2 |  | 4 |  | 1 |
| 3 | Match between system and real world | 3 | 2 |  |  | 5 |  | 2 | 2 | 1 |
| 4 | Consistency and Standards | 4 | 1 |  | 4 | 1 |  | 4 |  | 1 |
| 5 | Error Prevention | 3 | 2 |  | 3 | 2 |  | 3 | 1 | 1 |
| 6 | Help users recognize, diagnose, and recover from errors | 3 | 2 |  | 3 | 2 |  | 4 |  | 1 |
| 7 | Recognition rather than recall | 4 | 1 |  | 4 | 1 |  | 3 | 1 | 1 |
| 8 | Flexibility and efficiency of use | 2 | 3 |  | 4 | 1 |  | 3 | 1 | 1 |
| 9 | Aesthetic and minimalist design | 3 | 1 | 1 | 2 | 2 | 1 | 2 | 2 | 1 |
| 10 | Help and documentation^*^ | 3 | 1 |  | 2 | 3 |  |  | 4 | 1 |
| 11 | Privacy | 5 |  |  | 3 | 2 |  | 4 |  | 1 |
| 12 | Skills^*, #^ |  | 2 | 1 |  | 3 | 1 |  | 2 | 3 |
| 13 | Pleasurable interaction | 4 | 1 |  | 2 | 3 |  | 4 |  | 1 |
| 14 | Accessibility | 2 | 3 |  | 3 | 2 |  | 4 |  | 1 |
| 15 | Compatibility between different platforms |  | 4 | 1 |  | 4 | 1 | 3 | 1 | 1 |
| 16 | Minimized human/device interaction |  | 4 | 1 | 2 | 3 |  | 3 | 1 | 1 |
| 17 | Physical interaction and ergonomics^*^ | 2 | 2 |  | 2 | 3 |  | 3 | 1 | 1 |
| 18 | Readability and layout | 3 | 2 |  | 2 | 3 |  | 3 | 1 | 1 |
| 19 | Non-interruptive app information visualization | 3 | 2 |  | 4 | 1 |  | 3 | 1 | 1 |
| 20 | Content^#^ | 2 | 1 | 1 | 4 |  |  | 3 |  | 2 |
| 21 | Display | 2 | 3 |  | 4 | 1 |  | 3 |  | 2 |
| 22 | Navigation | 3 | 2 |  | 4 | 1 |  | 4 |  | 1 |
| 23 | Interactivity | 3 | 2 |  | 3 | 2 |  | 4 |  | 1 |
| 24 | Behavior change^#^ | 2 | 2 |  | 2 | 2 |  |  | 3 | 2 |
| 25 | Self-monitoring of blood glucose | 3 | 1 | 1 | 3 | 1 | 1 | 2 | 1 | 2 |
|  | **Total** | **113** | | **6** | **117** | | **5** | **93** | | **32** |

*Note. VI=Very Important; IM=Important; MI=Moderately Important; VR=Very Relevant; RE=Relevant; MR=Moderately Relevant; VG=Very Good; GD=Good; BA=Barely Acceptable.*
