# Supplementary material for: New Checklist for the Heuristic Evaluation of mHealth Apps (HE4EH): Development and Usability Study
Source: JMIR Mhealth Uhealth. 2020 Oct 28;8(10):e20353. doi: 10.2196/20353 (PMC7657716; doi:10.2196/20353)
Supplement: Multimedia Appendix 4 [file mhealth_v8i10e20353_app4.docx]

Mean score of the checklist items, as scored by the experts.

| **Question type** | **Mean score** | **Checklist items** |
| --- | --- | --- |
| **Clarity** | **2.8-2.99** | 3.26, 17.4 |
|  | **3.0-3.19** | 16.2, 18.6, 24.1 |
|  | **3.2-3.39** | 2.17, 12.3, 13.11, 13.9, 16.3, 16.5, 16.6, 19.1, 19.2, 24.11, 24.2, 24.22, 24.24, 24.25, 25.2, 25.24 |
|  | **3.4-3.59** | 1.1, 1.19, 1.2, 1.21, 1.28, 1.31, 1.5, 2.15, 2.16, 2.19, 4.38, 4.4, 4.6, 5.1, 7.3, 11.3, 12.2, 12.5, 12.6, 13.1, 13.7, 17.1, 18.7, 24.15, 24.23, 24.4, 24.5 |
|  | **3.6-3.79** | 1.27, 2.1, 2.13, 2.4, 2.5, 3.2, 4.29, 4.31, 4.32, 4.33, 4.34, 4.36, 4.5, 7.21, 8.11, 8.13, 8.15, 9.2, 11.1, 11.2, 11.5, 12.4, 13.1, 13.15, 13.2, 13.4, 13.5, 13.6, 15.1, 15.2, 16.7, 17.2, 17.5, 18.1, 18.4, 21.4, 23.2, 24.12, 24.14, 24.16, 24.17, 25.23, 25.32, 25.34, 25.6, 25.64 |
|  | **3.8-3.99** | 1.11, 1.17, 1.22, 1.25, 1.3, 1.33, 1.36, 1.9, 2.3, 4.12, 4.2, 4.22, 4.23, 4.27, 4.7, 4.8, 4.9, 5.1, 5.12, 5.13, 5.3, 5.9, 7.2, 7.23, 7.25, 7.27, 8.14, 8.16, 8.17, 8.19, 8.2, 9.1, 9.12, 9.14, 9.25, 9.28, 10.1, 10.3, 11.4, 12.1, 12.7, 13.12, 13.13, 13.16, 13.19, 13.2, 14.1, 16.1, 16.4, 18.2, 18.3, 18.5, 20.1, 21.1, 23.1, 23.3, 23.4, 24.18, 24.19, 24.2, 24.3, 24.6, 24.7, 24.9, 25.11, 25.17, 25.18, 25.2, 25.26, 25.28, 25.35, 25.37, 25.39, 25.4, 25.4, 25.41, 25.51, 25.61, 25.63, 25.65, 25.66 |
|  | **4.0-4.19** | 1.12, 1.13, 1.15, 1.16, 1.18, 1.29, 1.3, 1.35, 1.4, 1.6, 1.7, 1.8, 2.1, 2.11, 2.2, 2.21, 3.16, 4.1, 4.19, 4.2, 4.21, 4.24, 4.26, 4.3, 4.35, 4.4, 5.8, 7.1, 7.14, 7.28, 7.7, 7.8, 8.1, 8.12, 8.18, 8.23, 8.5, 9.11, 9.13, 9.15, 9.26, 9.32, 9.33, 9.5, 10.2, 10.22, 11.6, 13.14, 13.3, 13.8, 17.3, 19.3, 20.11, 20.5, 21.3, 21.5, 21.6, 24.1, 24.13, 24.26, 25.1, 25.13, 25.14, 25.15, 25.19, 25.22, 25.25, 25.3, 25.3, 25.31, 25.33, 25.36, 25.43, 25.44, 25.5, 25.6, 25.9, 1.1 |
|  | **4.2-4.39** | 1.2, 1.23, 2.12, 2.14, 2.18, 2.2, 2.22, 2.7, 2.9, 3.1, 3.12, 3.14, 3.18, 3.19, 3.2, 3.24, 3.4, 4.1, 4.13, 4.14, 4.16, 4.18, 4.25, 4.28, 4.3, 4.39, 5.14, 5.16, 5.2, 5.6, 5.7, 6.4, 7.12, 7.17, 7.19, 7.2, 7.26, 7.6, 8.21, 8.6, 8.7, 8.9, 9.16, 9.18, 9.2, 9.22, 9.23, 9.27, 9.29, 9.31, 9.35, 9.8, 10.18, 10.2, 20.7, 21.2, 22.5, 24.21, 24.8, 25.1, 25.12, 25.21, 25.27, 25.29, 25.38, 25.42, 25.45, 25.5, 25.53, 25.59, 25.7, 25.8 |
|  | **4.4-4.59** | 1.24, 1.26, 1.32, 1.34, 2.23, 2.6, 3.1, 3.11, 3.13, 3.15, 3.21, 3.22, 3.3, 3.5, 3.8, 4.11, 4.17, 4.37, 5.11, 5.15, 5.4, 5.5, 7.1, 7.11, 7.13, 7.15, 7.16, 7.18, 7.22, 7.24, 7.29, 7.4, 7.5, 8.1, 8.22, 8.4, 9.21, 9.24, 9.3, 9.3, 9.34, 9.36, 9.4, 9.6, 9.7, 9.9, 10.11, 10.12, 10.14, 10.16, 10.17, 10.21, 10.4, 10.7, 10.8, 10.9, 13.17, 20.1, 20.3, 20.4, 20.6, 20.8, 22.2, 22.4, 22.6, 22.7, 22.8, 25.16, 25.47, 25.49, 25.52, 25.54, 25.55, 25.58 |
|  | **4.6-4.79** | 1.14, 2.8, 3.17, 3.6, 3.7, 3.9, 4.15, 6.1, 6.2, 7.3, 7.9, 8.2, 8.3, 8.8, 9.1, 9.17, 9.19, 10.1, 10.15, 10.19, 10.5, 10.6, 13.18, 19.4, 20.9, 22.1, 22.3, 25.46, 25.48, 25.56, 25.57, 25.62 |
|  | **4.8-5.0** | 3.23, 3.25, 6.3, 10.13, 20.2 |
| **Grouping** | **2.8-2.99** | None |
|  | **3.0-3.19** | 2.19, 8.22, 19.3, 24.1, 25.2 |
|  | **3.2-3.39** | 1.35, 2.17, 4.36, 4.8, 9.2, 13.1, 16.6, 16.7, 17.2, 18.6, 22.7, 24.22, 24.25 |
|  | **3.4-3.59** | 1.13, 1.21, 2.14, 2.3, 4.13, 4.18, 4.32, 8.13, 9.36, 10.18, 11.5, 13.12, 16.2, 16.3, 16.5, 18.1, 21.6, 23.1, 24.11, 24.18, 24.24, 24.26, 24.5, 25.18, 25.59, 25.63 |
|  | **3.6-3.79** | 1.12, 1.24, 1.25, 1.31, 1.4, 2.21, 2.4, 2.5, 3.11, 4.1, 4.22, 4.25, 4.34, 4.38, 4.6, 4.7, 7.21, 7.23, 9.23, 9.27, 10.22, 11.1, 12.3, 12.4, 12.6, 13.11, 13.15, 17.5, 18.4, 20.1, 20.4, 22.6, 23.3, 24.16, 24.2, 24.8, 25.11, 25.13, 25.2, 25.26, 25.34, 25.52, 25.64, 25.65, 25.8 |
|  | **3.8-3.99** | 1.1, 1.11, 1.29, 1.5, 1.6, 1.8, 2.11, 2.2, 2.23, 2.6, 3.2, 3.23, 3.26, 4.21, 4.27, 4.29, 4.3, 4.31, 4.4, 5.8, 7.19, 7.27, 8.1, 8.14, 8.17, 8.19, 8.23, 8.9, 13.1, 13.13, 13.3, 13.4, 15.2, 16.1, 17.1, 17.3, 17.4, 18.2, 18.5, 20.6, 21.1, 21.2, 21.4, 22.5, 24.15, 24.23, 24.9, 25.15, 25.23, 25.24, 25.25, 25.29, 25.3, 25.32, 25.4, 25.48, 25.5, 25.53, 25.57, 25.66 |
|  | **4.0-4.19** | 1.18, 1.19, 1.23, 1.28, 1.3, 1.32, 1.9, 2.1, 2.12, 2.15, 2.2, 2.22, 3.12, 3.18, 3.19, 3.6, 3.7, 4.11, 4.17, 4.2, 4.26, 4.3, 4.33, 4.35, 5.1, 5.14, 5.2, 6.2, 7.26, 7.28, 7.29, 8.2, 9.33, 9.9, 10.1, 10.1, 10.12, 10.16, 10.21, 10.3, 11.3, 12.1, 12.2, 12.5, 12.7, 13.17, 13.2, 13.5, 13.7, 13.9, 15.1, 16.4, 18.7, 19.1, 19.2, 20.1, 20.2, 21.3, 21.5, 21.7, 22.2, 22.3, 22.4, 23.4, 24.1, 24.12, 24.13, 24.17, 24.2, 24.21, 24.3, 24.4, 25.12, 25.14, 25.16, 25.17, 25.19, 25.21, 25.36, 25.43, 25.5, 25.51, 25.9 |
|  | **4.2-4.39** | 1.1, 1.15, 1.2, 1.22, 1.27, 1.33, 1.36, 1.7, 2.16, 2.18, 2.7, 2.9, 3.15, 3.24, 3.4, 3.5, 3.9, 4.12, 4.14, 4.16, 4.19, 4.2, 4.23, 4.24, 4.39, 4.4, 4.5, 5.13, 5.15, 5.3, 5.4, 5.7, 7.12, 7.2, 7.22, 7.24, 7.25, 7.3, 8.11, 8.12, 8.21, 8.4, 8.5, 8.6, 9.1, 9.12, 9.17, 9.2, 9.24, 9.34, 10.11, 10.14, 10.19, 10.9, 11.4, 13.2, 13.8, 14.1, 18.3, 19.4, 20.3, 20.8, 24.14, 24.6, 24.7, 25.22, 25.27, 25.33, 25.37, 25.39, 25.4, 25.44, 25.45, 25.46, 25.56, 25.6, 25.6, 25.7 |
|  | **4.4-4.59** | 1.14, 1.17, 1.2, 1.26, 2.1, 2.13, 3.13, 3.14, 3.17, 3.2, 3.21, 3.22, 3.3, 3.8, 4.1, 4.15, 4.28, 4.37, 4.9, 5.11, 5.16, 5.5, 5.6, 5.9, 6.1, 7.1, 7.11, 7.17, 7.18, 7.4, 7.6, 8.1, 8.3, 8.7, 8.8, 9.1, 9.16, 9.18, 9.19, 9.25, 9.28, 9.29, 9.31, 9.4, 9.5, 9.6, 10.2, 10.4, 10.6, 11.2, 11.6, 13.14, 13.16, 13.19, 13.6, 20.5, 20.7, 20.9, 22.1, 22.8, 23.2, 24.19, 25.1, 25.1, 25.28, 25.3, 25.31, 25.35, 25.41, 25.47, 25.54, 25.55, 25.61, 25.62 |
|  | **4.6-4.79** | 1.3, 1.34, 2.8, 3.1, 3.1, 5.1, 5.12, 6.4, 7.14, 7.2, 7.3, 7.5, 7.8, 8.15, 8.16, 9.13, 9.14, 9.15, 9.21, 9.22, 9.26, 9.3, 9.3, 9.35, 9.8, 10.13, 10.15, 10.17, 10.2, 10.5, 10.8, 13.18, 25.58 |
|  | **4.8-5.0** | 1.16, 3.16, 3.25, 6.3, 7.1, 7.13, 7.15, 7.7, 7.9, 8.18, 8.2, 9.11, 9.32, 10.7, 20.11, 25.38, 25.42, 25.49, 7.16, 9.7 |
| **Relevance** | **2.8-2.99** | 12.3, 17.4 |
|  | **3.0-3.19** | 11.1 |
|  | **3.2-3.39** | 2.22, 3.26, 4.13, 4.27, 13.12, 17.1, 18.2, 18.3, 23.4, 25.25 |
|  | **3.4-3.59** | 1.6, 2.16, 2.4, 2.6, 4.17, 4.21, 12.6, 13.16, 16.2, 17.2, 18.1, 18.5, 18.6, 19.1, 19.2, 23.3, 24.2, 24.22, 24.4, 25.13, 25.23 |
|  | **3.6-3.79** | 1.2, 1.22, 1.35, 1.5, 2.18, 2.19, 2.3, 3.7, 4.11, 4.18, 4.19, 4.2, 4.23, 4.29, 4.3, 5.3, 8.12, 8.22, 9.1, 9.17, 9.19, 9.21, 10.22, 11.2, 12.2, 12.4, 13.1, 13.2, 14.1, 15.2, 16.4, 16.7, 17.3, 18.7, 20.1, 20.8, 21.2, 22.4, 22.7, 24.11, 24.12, 24.16, 24.17, 24.26, 24.8, 25.24, 25.3, 25.31, 25.35, 25.38, 25.4, 25.54, 25.62, 25.64 |
|  | **3.8-3.99** | 1.1, 1.12, 1.14, 1.2, 1.27, 1.7, 1.8, 2.12, 2.21, 4.15, 4.16, 4.25, 4.33, 4.34, 4.38, 4.8, 4.9, 5.11, 5.15, 8.19, 9.28, 9.3, 9.9, 11.3, 11.4, 11.5, 12.1, 13.1, 13.11, 13.13, 13.3, 13.4, 13.7, 13.8, 16.3, 19.3, 20.3, 20.6, 21.6, 21.7, 22.3, 22.8, 23.2, 24.13, 24.15, 24.19, 24.21, 24.25, 25.17, 25.19, 25.2, 25.48, 25.53, 25.56, 25.6 |
|  | **4.0-4.19** | 1.16, 1.21, 1.26, 1.28, 1.29, 1.36, 2.11, 2.15, 2.2, 2.2, 2.5, 2.8, 3.19, 3.5, 4.1, 4.1, 4.22, 4.26, 4.28, 4.31, 4.37, 4.5, 4.6, 4.7, 5.1, 5.13, 5.5, 7.3, 8.1, 8.14, 8.18, 8.8, 9.11, 9.12, 9.13, 9.14, 9.15, 9.16, 9.23, 9.26, 9.3, 9.34, 9.5, 10.19, 10.21, 11.6, 12.5, 13.6, 15.1, 16.1, 16.5, 16.6, 17.5, 18.4, 20.2, 20.9, 21.1, 21.3, 24.1, 24.14, 24.18, 24.23, 24.3, 24.6, 25.1, 25.14, 25.16, 25.18, 25.21, 25.27, 25.29, 25.32, 25.39, 25.45, 25.66, 25.7, 25.8 |
|  | **4.2-4.39** | 1.11, 1.17, 1.24, 1.25, 1.3, 1.32, 1.34, 2.1, 2.14, 2.17, 3.12, 4.12, 4.14, 4.2, 4.3, 4.32, 4.35, 4.39, 4.4, 5.14, 5.4, 7.14, 7.18, 7.2, 7.25, 7.28, 7.5, 7.7, 8.11, 8.16, 8.4, 8.5, 9.1, 9.2, 9.29, 9.6, 10.16, 10.17, 10.18, 10.2, 12.7, 13.14, 13.15, 13.2, 13.5, 13.9, 19.4, 22.1, 22.5, 24.1, 24.24, 24.5, 24.7, 25.1, 25.11, 25.12, 25.2, 25.22, 25.26, 25.3, 25.33, 25.41, 25.44, 25.46, 25.5, 25.5, 25.52, 25.57, 25.58, 25.6, 25.63 |
|  | **4.4-4.59** | 1.1, 1.15, 1.18, 1.19, 1.31, 1.33, 2.1, 2.7, 3.1, 3.11, 3.13, 3.15, 3.21, 3.22, 3.3, 3.9, 4.24, 4.36, 4.4, 5.1, 5.2, 5.8, 6.2, 7.11, 7.15, 7.16, 7.17, 7.22, 7.23, 7.24, 7.29, 7.3, 7.9, 8.13, 8.17, 8.2, 9.22, 9.24, 9.25, 9.32, 9.4, 9.7, 9.8, 10.1, 10.11, 13.17, 20.4, 21.4, 21.5, 22.2, 23.1, 25.15, 25.28, 25.34, 25.4, 25.42, 25.47, 25.51, 25.59, 25.61, 25.9 |
|  | **4.6-4.79** | 1.13, 1.3, 1.4, 1.9, 2.13, 2.9, 3.14, 3.16, 3.17, 3.2, 3.25, 3.6, 5.12, 5.7, 5.9, 6.1, 6.3, 7.12, 7.13, 7.19, 8.15, 8.2, 8.3, 8.6, 8.9, 9.18, 9.2, 9.27, 9.31, 9.33, 9.35, 9.36, 10.1, 10.15, 10.3, 10.5, 10.7, 13.18, 13.19, 20.1, 20.11, 20.7, 22.6, 24.2, 24.9, 25.36, 25.37, 25.43, 25.49, 25.55, 25.65 |
|  | **4.8-5.0** | 1.23, 3.1, 3.18, 3.2, 3.23, 3.24, 3.4, 3.8, 7.1, 7.1, 7.2, 7.21, 7.26, 7.4, 7.6, 7.8, 8.1, 8.21, 8.23, 8.7, 10.12, 10.13, 10.4, 10.8, 10.9, 20.5, 2.23, 5.16, 5.6, 6.4, 7.27, 10.14, 10.2, 10.6 |
