# Supplementary material for: New Checklist for the Heuristic Evaluation of mHealth Apps (HE4EH): Development and Usability Study
Source: JMIR Mhealth Uhealth. 2020 Oct 28;8(10):e20353. doi: 10.2196/20353 (PMC7657716; doi:10.2196/20353)
Supplement: Multimedia Appendix 6 [file mhealth_v8i10e20353_app6.docx]

Severity rating scale 2.

| **Rating** | **Classification** | **Mean** | **Overall mean** |
| --- | --- | --- | --- |
| 0 | NOT APPLICABLE | 4.0 | 3.6 |
| 1 | NO VIOLANCE | 3.2 |  |
| 2 | MINOR | 4.2 |  |
| 3 | MAJOR | 3.4 |  |
| 4 | CATASTROPHE | 3.2 |  |
