# Supplementary material for: New Checklist for the Heuristic Evaluation of mHealth Apps (HE4EH): Development and Usability Study
Source: JMIR Mhealth Uhealth. 2020 Oct 28;8(10):e20353. doi: 10.2196/20353 (PMC7657716; doi:10.2196/20353)
Supplement: Multimedia Appendix 8 [file mhealth_v8i10e20353_app8.docx]

Participants’ comments about issues with the heuristics.

| **HE** | **Problem description** | **Potential solution** | **SR** |
| --- | --- | --- | --- |
| 3 | Too much navigation is needed if, for example the user needs to analyze the patterns of his glucose readings. The patterns icon allows access to the patterns easily, but only if the user inserts several inputs | Make the patterns accessible and viewable in the dashboard instead of inside nested menus | 2 |
| 6 | If the username is correct, but the password is incorrect, user still gets a message “Username or passwords are incorrect.” | A specific message stating password is incorrect would be more meaningful and logical. | 2 |
| 7 | In the goal setting screen (under My settings), the measurement and entry instructions (if any) of the data to be entered must be clearly stated. For Example, for the “carb entries”, when enter 20. You get a message that says you need to enter a number between 1 and 10. | It would have been better for the user to see this instruction prior to entering the data | 3 |
| 11 | The app keeps the username after the logout. There is no option for the user to remove the username information or even for another user to login with its own credentials. | There should be an option for the user to change username. | 3 |
| 16 | Hard to scroll and find country. | Prefer a search box instead | 3 |
| 20 | There is no indication for the minimum and maximum value of A1C. By applying different values, you get an indication that the minimum value allowed is 5, while the maximum value allowed is 13.5. | Both the minimum and the maximum should be shown to user just below the textfield. | 2 |
| 22 | Label are not visible and in cases not available | Include label where needed to guide the user and simplify navigating the application | 3 |
| 24 | I couldn’t find an icon that instructs me to share with others or to buddy with individuals to promote behavioral change and encourage me to stay up to track for adequate monitoring. Especially for a conservative community such as Qatar, it requires higher level of consideration for this matter. | Maybe including a group chat “monthly” for example for others who have kids with diabetes to stay up to date and have an update about their performance. This might promote behavioral change and to be able to have conversations with individuals with similar conditions. | 3 |
| 25 | The app does not inform user about the frequency of glucose reading and how often the sensor is scanned. | The app can update user about the frequency of glucose reading and the frequency of reading through the scanner | 2 |
| OT | Expected the share option to include social media sharing via link to report on WhatsApp |  | 2 |
| OT | The app only shows the recent A1C in the chart even though multiple A1C values are entered in the app. | Either all should be shown with different colored lines and clicking on any line shows the information | 3 |

*Note. HE=heuristic number; OT=guideline was not present in the given set; SR=severity rating.*
